# Cues for different diagnostic patterns of interpersonal violence in a psychiatric sample: an observational study

**DOI:** 10.1186/s12888-020-02594-0

**Published:** 2020-05-01

**Authors:** Dalila Talevi, Alberto Collazzoni, Alessandro Rossi, Paolo Stratta, Monica Mazza, Francesca Pacitti, Manuela Costa, Claudio Crescini, Rodolfo Rossi

**Affiliations:** 1 Department of Biotechnological and Applied Clinical Sciences (DISCAB), University of L’Aquila, L’Aquila, Italy; 2 Department of Mental Health, ASL 1 Avezzano Sulmona L’Aquila, L’Aquila, Italy; 3 Chair of Psychiatry, Department of Systems Medicine, University of Rome Tor Vergata, Rome, Italy

**Keywords:** Interpersonal violence, Mental illness, Gender, Young age, Psychosis, Mood disorders, Personality disorders

## Abstract

**Background:**

Interpersonal violence has increased as a health concern, especially in psychiatry practice, over the last decades. Nevertheless, most patients with stable mental disorders do not present an increased risk of violence, and mental disorder is not a necessary or sufficient cause of violent behaviours. People with mental disorders endorse more often a number of risk factors for violence that could confound this association, such as young age and male gender. The aim of this study was to investigate the effect of age, gender, and diagnosis on reported levels of interpersonal violence in a sample of people with severe mental illness.

**Methods:**

The sample was composed of 160 inpatients: 73 with a psychosis within the schizophrenia spectrum, 53 with a mood disorder and 34 with a personality disorder. All patients enrolled in the study were assessed for experiences of victimization and perpetration of interpersonal violence using the Karolinska Interpersonal Violence Scale interview. Demographic variables were also collected.

**Results:**

Both violence perpetration and victimization negatively correlated with age. Compared to males, females were exposed to higher degree of victimization in childhood and adulthood, whereas males were more involved in the perpetration of violence in childhood. Personality disorders were associated with higher levels of interpersonal violence, both perpetration and victimization; an interaction effect of gender and diagnosis was also observed for violence perpetration in adulthood. Distinct patterns of interpersonal violence did emerge for the diagnostic groups with mood disorder showing a victimization pattern, personality disorders a perpetration pattern and psychoses less defined patterns.

**Conclusions:**

The main finding is that psychotic disorders, mood disorders and personality disorders have different patterns of violent experiences interacting with age and gender. This study offers a better understanding of how gender and age could affect violent behaviours. Moreover, study findings may increase the comprehension of the reason why some mental disorders, compared to others, are more associated with the risk of victimization or perpetration of violence. These patterns could have pathophysiological or pathoplastic meaning addressing clinical and diagnostic trajectories and they could interact with other intervening risk factors.

## Background

Interpersonal violence (IV) has become a major public health issue over the last decades [1], being a significant cause of morbidity and mortality worldwide [2, 3]. IV has increased as a health concern especially in psychiatry practice [4]. IV encompasses two main components: expression (i.e., perpetration) of and exposure (i.e., victimization) to violence. It’s important to distinguish victimization and perpetration experiences given that the two types of violence are associated with different risk factors, health consequences and management [5].

Most patients with stable mental disorders do not present an increased risk of violent behaviour [6] and they are more likely to be victims than perpetrators [7–9]. However, schizophrenia, mood disorders, post-traumatic stress disorder, personality disorders and substance use disorders are related to a high occurrence of violent behaviours especially when active symptoms or a relapse are present [6, 8, 10–13]. A broad body of research focused on the detection of risk factors for violence perpetration related to psychiatric morbidity. Most epidemiological and clinical studies support the notion that mental disorders provide a modest contribution to violence risk among adults [11, 14, 15], concluding that a mental disorder is not a necessary or sufficient cause of violent behaviours [7], nor an independent predictor [16]. Patients who more frequently reported violent experiences showed other factors associated with violence, such as historical, dispositional, and contextual ones [6, 14, 16].

Among demographic variables associated with violence, age and gender have received large attention so far [2, 11, 17]. Literature consistently reports that younger age is associated with high levels of both perpetration and victimization in community samples as well as clinical and forensic populations [2, 7, 9, 11, 12, 16–20]. The role of gender in IV is largely debated and controversial, with both evidence supporting gender imbalance in violence perpetration (i.e., males > females, [6, 7, 11, 16]; females > males, [12]), as well as studies reporting similar rates in males and females [21–24]. The gender pattern of victimization is more consistent, with women being overly victimized compared to men [8, 9, 12, 21, 24–28]. Globally, evidence suggests that men represent the majority of perpetrators and women the majority of victims [29].

### Aims and hypotheses

Suffering from a severe mental illness (SMI) might increase the probability of experiencing violence; however, people with SMI endorse more often a number of risk factors for violence that could confound this association.

The goal of this study is to investigate how the diagnosis of a SMI interact with age and gender in the context of violent experiences. We aimed to detect any diagnosis-life stages and diagnosis-gender pattern associated with violence perpetration and victimization. The Karolinska Interpersonal Violence Scale (KIVS) was used as it measures expression of and exposure to violence, consistent with a victimization and a perpetration pattern of interpersonal violence, respectively. Our hypothesis is that different victimization/perpetration patterns exist for psychoses within the schizophrenia spectrum and other psychotic disorders (SSOPD), mood disorders and personality disorders when combining diagnosis with age and with gender.

## Methods

### Study design

This study had a cross-sectional observational design. Data were collected at the psychiatric unit of the L’Aquila San Salvatore Hospital, Italy, in collaboration with the Department of Biotechnological and Applied Clinical Sciences of University (DISCAB) from December 2016 to March 2018. All the procedures and the research project were approved by the local ethics committee.

### Participants and procedures

Among 230 consecutively admitted patients who were eligible for the study, 164 agreed to participate (71.3% of eligible patients); 66 (28.7% of eligible patients) refused to participate for many reasons. Our final sample was of 160 patients (69.6% of eligible patients) hospitalized for a SMI (i.e., mood disorders, SSOPD, and personality disorders) index episode. Four patients showed more than 5% of missing data and were therefore not involved in statistical computations.

The primary psychiatric diagnosis was established by senior psychiatrists (AR, FP), according to the Diagnostic and Statistical Manual of Mental Disorders 5^th^ edition [30] criteria. Participants were appropriately informed of the possibility of being recruited into the study through clinical interview and information materials posted on the bulletin boards of the ward. Each patient gave written informed consent prior to inclusion. Subjects who provided consent were interviewed for socio-demographic characteristics and evaluated for assessing violence perpetration and victimization experiences. Evaluations were performed when patients achieved remission, in order to minimize bias due to variations of clinical conditions. Exclusion criteria were: a) age > 65 years, b) language barriers, c) impaired consciousness, d) severe aphasia, and e) intellectual disability or other cognitive deficits.

### Assessment tool for interpersonal violence

Violence victimization and perpetration were assessed using the KIVS. KIVS is composed of four rating scales assessing exposure to violence (“victim of violence” subscales) and expressed violence behaviour (“used violence” subscales) in childhood (between 6 and 14 years of age) and adulthood (from 15 years upwards). The steps of the KIVS are defined by short statements about concrete examples of violent episodes of increasing severity and frequency that could have occurred throughout the respondent’s lifetime. The ratings (0–5 for each subscale, in total maximum of 20) are based on a semi-structured interview performed by trained clinicians. KIVS is specific for IV and distinguished aggressive acts from thoughts. It was validated against several questionnaires measuring aggression and acts of violence and has good psychometric properties [31]. Moreover, it allows for use of composite scores of its subscales [32]. It has been used in several suicide research studies [19, 33–36] and in observational studies within clinical samples [32, 37–40]. In the current study, we used the four subscales separately as well as the composite scores of lifetime (from childhood to adulthood) expressed violence and exposure to violence (“lifetime expressed violent behaviour” composite score and “lifetime exposure to violence” composite score). Upon completion of a back-translation process, the Italian version was administered after authorization by original authors [41].

### Statistical analysis

Data were analyzed using the Statistical Package for Social Sciences (SPSS^®^, version 24, IBM, U.S.A.). Mean ± SD, and frequencies were calculated for descriptive analysis. Diagnostic differences in violence experiences were examined using a one-way analysis of variance (ANOVA) with Bonferroni’s post hoc test among the three diagnostic groups.

In order to investigate gender differences on levels of violence, an independent-samples *t*-test was calculated for all the KIVS subscales. *t*-test was initially performed on the whole sample, and subsequently it was repeated stratifying the sample by diagnosis and gender.

Bivariate Pearson correlations were calculated between age and the composite KIVS scores of lifetime expressed violence and exposure to violence.

Two-way ANOVAs were conducted to test the diagnosis-gender pattern of perpetration and victimization.

Finally, to test the diagnosis-life stages pattern, bivariate Pearson correlations was calculated between the four KIVS subscales.

## Results

### Descriptive characteristics

The sample was composed of 86 male patients (53.80%) and 74 (46.30%) female patients, with a mean age of 41.15 ± 12.60 years. About 90% of the participants were of Italian nationality and almost 99% were Caucasians. Most of them belonged to the lower class, had a medium level of education (secondary or high school, 76.20%), were single (60.60%) and unemployed (48.80%). Seventy-three subjects were diagnosed as affected by SSOPD (45.6%), 53 by mood disorders (33.1%), and 34 by personality disorders (21.3%; Table 1).

**Table 1:** Demographic characteristics of the sample (*N* = 160)

		Mean (SD)/n (%)
**Age**		41.15 (12.60)
**Marital status**	Single	97 (60.6)
	Married/cohabitant	31 (19.4)
	Separated/divorced	32 (20)
**Education level**	Low education	7 (4.4)
	Medium education	122 (76.2)
	High education	31 (19.4)
**Occupational status**	Unemployed	78 (48.8)
	Employed	44 (27.5)
	Others	38 (23.7)

*Note*: Low education refers to illiteracy and primary school certificate; medium education refers to secondary and high school certificate; high education refers to graduation and post-graduate degree. Others (Occupational status) refers to odd jobs, pensioners, students and housewives

The KIVS mean scores in the total sample were: Total score = 4.91 ± 3.57; Used violence as a child subscale = 0.48 ± 0.84; Used violence as an adult subscale = 1.03 ± 1.28; Victim of violence in childhood subscale = 1.81 ± 1.59; Victim of violence in adulthood subscale = 1.61 ± 1.47.

Incidentally, even though it was not a primary aim of the study, KIVS differences between the clinical sample and a matched control group (*n* = 160) was calculated. The control group showed lower scores for all the KIVS subscales (Total score = 1.16 ± 0.80; Used violence as a child subscale = 0.22 ± 0.63; Used violence as an adult subscale = 0.12 ± 0.50; Victim of violence in childhood subscale = 0.44 ± 0.32; Victim of violence in adulthood subscale = 0.37 ± 0.26). All these differences were significant (two-tailed independent t-test at the 5% level, *p* < .001). The same results were confirmed in an expanded sample [41].

### Diagnostic, age and gender differences

The personality disorders group had higher Used violence as an adult [F (2, 157) = 3,64, *p* = .03], Victim of violence in adulthood [F (2, 157) = 5.05, *p* = .01], and KIVS total score [F (2, 157) = 4,96, *p* = .01] values than the patients with mood disorders. No other significant differences were found between the groups (Table 2).

**Table 2:** One-way ANOVA analysis evaluating the differences on KIVS subscales and total score in the three diagnostic groups (Mean + SD)

Variables (KIVS scores)	**SSOPD (1)** (*n* = 73)	**Mood Disorders (2)** (*n* = 53)	**Personality Disorders (3)** (*n* = 34)	**F**	**Post hoc comparison**^**a**^
Used violence as a child	0.48 (0.88)	0.34 (0.68)	0.68 (0.94)	1.69	–
Used violence as an adult	1.01 (1.22)	0.75 (1.11)	1.5 (1.54)	3.64^*^	3 > 2^*^
Victim of violence in childhood	1.77 (1.41)	1.72 (1.70)	2.06 (1.79)	0.53	–
Victim of violence in adulthood	1.63 (1.37)	1.21 (1.20)	2.21 (1.84)	5.05^**^	3 > 2^**^
Total score	4.88 (3.21)	4 (3.27)	6.41 (4.29)	4.96^**^	3 > 2^**^

*Note*. SSOPD = schizophrenia spectrum and other psychotic disorders; KIVS = Karolinska Interpersonal Violence Scale

^*^p < .05 ^**^p < .01

^a^ Bonferroni post hoc

In the total sample, violence (KIVS total score) negatively correlated with age (*r* = −.33), both victimization (“lifetime exposure to violence” composite score; *r* = −.28) and perpetration (“lifetime expressed violent behaviour” composite score; *r* = −.27).

Used violence as a child score is higher in males (0.64 ± 0.90) than in females (0.28 ± 0.71), [t (158) = 2.77, *p* = .01]. Victim of violence in childhood score is higher in females (2.08 ± 1.74) than in males (1.58 ± 1.41), [t (158) = − 1.97, *p* = .05] as well as Victim of violence in adulthood score (females: 1.92 ± 1.67; males: 1.35 ± 1.21), [t (158) = − 2.43, *p* = .02]. No significant differences were found for Used violence as an adult (females: 0.89 ± 1.21; males: 1.15 ± 1.33) and KIVS total score (females: 5.16 ± 3.62; males: 4.70 ± 3.53).

### Diagnosis-life stages pattern

**Fig. 1:**
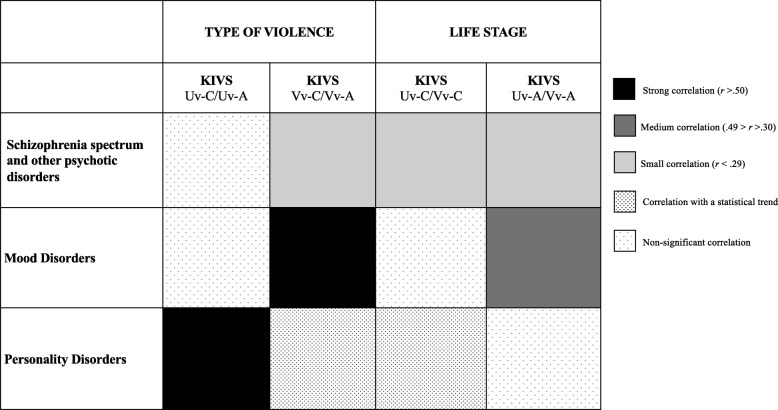
Diagnostic-life stages patterns of violence victimization and perpetration in Schizophrenia spectrum and other psychotic disorders, Mood and Personality Disorders^a^. *Note*. ^a^Pearson’s correlation coefficient at the .05 level of significance. KIVS = Karolinska Interpersonal Violence Scale; Uv-C = Used violence as a child; Uv-A = Used violence as an adult; Vv-C = Victim of violence in childhood; Vv-A = Victim of violence in adulthood

Significant and positive correlations between the different types of violence at two life stages (i.e., childhood and adulthood) in the three diagnostic groups have been found (Fig. 1).

The Victim of violence in childhood subscale showed a significant, positive, weak correlation with the Victim of violence in adulthood subscale in SSOPD patients (*r* = .24). Similarly, the Victim of violence in childhood and the Victim of violence in adulthood subscales showed a significant, positive, but higher, correlation in mood disorders sample (*r* = .52). On the contrary, in the personality disorder sample, the Used violence as a child and the Used violence as an adult subscales showed a significant, positive correlation (*r* = .57).

The Used violence as a child subscale had a significant, positive, but small correlation with the Victim of violence in childhood subscale only in the SSOPD sample (*r* = .25). Instead, the Used violence as an adult subscale is significantly and positively correlated to the Victim of violence in adulthood subscale in SSOPD (*r* = .29) and mood disorder (*r* = .37) samples.

### Diagnosis-gender pattern

**Fig. 2:**
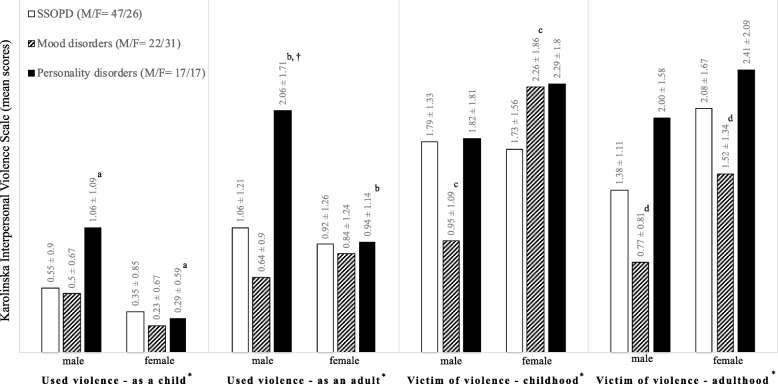
Diagnostic-gender patterns of violence victimization and perpetration in Schizophrenia spectrum and other psychotic disorders, Mood and Personality Disorders. *Note.* SSOPD = Schizophrenia spectrum and other psychotic disorders; M = males; F = females; M/F = distribution in males and females in each diagnostic group. Karolinska Interpersonal Violence Scale (KIVS) mean scores for diagnoses and genders were calculated. *independent samples *t*-test was used for testing statistically significant differences among males and females of each diagnostic group. ^a,b,c,d^ Significant differences at the .05 level. ^†^A Two-way analysis of variance (ANOVA) was conducted on the influence of gender and diagnosis on level of violence measured by each subscale of the Karolinska Interpersonal Violence Scale

The *t*-test, when the sample was broken down by diagnosis, showed significant gender differences for the KIVS subscales in mood and personality disorders groups, but not in SSOPD one (Fig. 2).

The two-way ANOVAs reported a statistically significant interaction between the effects of gender and diagnosis on Used violence as an adult subscale [F (2, 154) = 2.99, *p* = .05]. To better understand if the Used violence as an adult is different in the considered diagnoses for gender, we analyzed males and females separately. We ran a one-way ANOVA for just diagnoses on either group. The one-way ANOVA performed on Used violence as an adult subscale [F (2, 83) = 6.40, *p* = .03] in all of the three-diagnosis, showed that the male patients with a diagnosis of personality disorder had higher scores than the other patients. No differences were reported between the other diagnoses. Finally, no differences have been found in female group. There was not a statistically significant interaction between the effects of gender and diagnosis on Victim of violence in childhood [F (2, 154) = 2.76, *p* = .07], on Victim of violence in adulthood [F (2, 154), = .16, *p*=. 85), on Used violence as a child subscales [F (2, 154), = 1.40, *p* = .25], and on KIVS total score [F (2, 154), = 2.12, *p* = .12] (Fig. 2).

## Discussion

### Main findings

The main finding of this study is that psychotic disorders (SSOPD), mood disorders and personality disorders have different patterns of violent experiences when combined with age and gender.

Overall, in our sample, people with personality disorders showed the highest levels of both violence perpetration and victimization, reporting significant differences compared to mood disorders in adulthood. Younger age was associated with both perpetration and victimization. Females were more often victimized than males, both in childhood and adulthood, whereas males engaged more often in violent behaviours than females in early life. When we explored the episodes of violence victimization and perpetration that occurred in childhood and adulthood, a distinct diagnosis-life stages pattern did emerge for the three diagnostic groups, with mood disorders showing a strong victimization pattern, personality disorders a strong perpetration pattern and SSOPD less defined patterns. For people affected by a mood disorder, victimization in childhood is positively correlated with victimization in adulthood. Moreover, victimization was positively correlated with perpetration in adults with mood disorders, based on a medium “victimization-perpetration association pattern”. This finding is in line with the evidence that victimized subjects are more likely to engage in violent events [9, 12]. On the contrary, in personality disorders, engaging in violent behaviour in childhood is positively and strongly correlated with violent acts perpetrated in adulthood. People with SSOPD showed weaker “childhood-adulthood victimization association pattern” and “victimization-perpetration association pattern” (both childhood and adulthood) than other patients (see Fig. 1).

Our findings on the diagnosis-gender pattern showed that male subjects suffering from a personality disorder had higher scores than other patients in “Used violence as an adult subscale”. We could claim that in these subjects a cluster of risk factors (i.e., gender, marital status, diagnosis, substance misuse) interact with each other increasing exponentially the risk of violent behaviours.

According to a more general perspective, our findings about the gender-diagnosis interaction allows some comments. Firstly, the absence of the interaction in childhood was expectable: a psychiatric condition might not have developed or be pervasive yet at that time. Secondly, gender and diagnosis had an interaction effect only on expression of violence in adulthood, that was the only subscale not to show a gender difference, although showing differences among diagnostic groups. This finding could mean that a specific SMI in a specific gender might have a more pervasive impact on the perpetration of violence, not on victimization. These findings should be interpreted carefully given their inherent methodological shortcomings.

Males had higher scores in used violence subscales than females with the same diagnosis and in the same period of life; on the contrary, females had the highest scores on victimization subscales. This pattern did not repeat for “used violence as an adult” and “victim of violence in childhood”: in the first case, females with mood disorders reported greater expression of violence than males; in the second case, female victims of violence in childhood with a mood disorder got higher scores than females with SSOPD. Moreover, males with SSOPD reported higher levels of victimization in childhood then females. Although the results are not statistically significant in most cases, they and their graphic representations provide stimulating cues. For example, mood disorders showed to have a trend of a lower involvement in both violence perpetration and victimization, in particular for males; both males and females with personality disorders showed a trend of greater involvement in the expression of and in the exposure to violence, respectively (see Fig. 2).

### Previous literature

Overall, our findings are consistent with pre-existing literature. As regards the relation between mental disorders and violence, a recent large American population-based study confirms that personality disorders show higher odds for violence perpetration [11], compared to other mental disorders. Cluster B and paranoid personality disorders are considered the most likely linked to violent offending and aggression [42, 43], to suicidal behaviours and criminal arrest [44]. The association between personality disorders and violence perpetration is possibly linked to their intrinsic impulsiveness, substance abuse and bio-psychological mechanisms [6, 45]. As regards SSOPD, only a modest relation with violent acts has been found in several large population-based studies [46]; regarding mood disorders, some evidence suggests an increased risk of engaging in violence in particular those with bipolar disorder [47].

Our results regarding the correlation between age and violence are consistent with other researches [2, 9, 11, 15, 17, 19]. Using the KIVS, which distinguishes exposure to and expression of violence in childhood and adulthood, we demonstrated the strong, inverse correlation between age and both perpetration and victimization. Further studies are necessary to understand if the young are actually more violent than older people, or they merely tend to remember more often or disclose more freely violent episodes.

With regard to gender, the results concerning the victimization pattern are in line with previous literature reporting that females are the main victims of IV [8, 9, 12, 21, 25–28]. When broken down by diagnosis, the exposure to violence both in childhood and adulthood was more frequent in females for mood disorders only. This finding is somewhat in line with studies reporting an association between victimization and the presence of a mood disorder in females [8, 11, 27]. As regards perpetration of violence, we replicated that males are more likely than females to act aggressively in childhood [19, 31, 35, 48]. On the other hand, we did not find a significant difference for violence perpetration in adulthood between males and females, contrary to previous studies. This finding could be due to several reasons, including the small sample size. The largest epidemiological studies on this issue found that males commit acts of violence at greater rates than females [11, 14, 15, 49]; only the National Institute of Mental Health Clinical Antipsychotic Trials of Intervention Effectiveness (CATIE), which investigated violent outcomes in schizophrenia patients as part of a large multisite randomized clinical trial, found an association between female, rather than male gender, and minor violence [50]. A paucity of other studies came to different conclusions: in an extensive review, Gillies & Brien [18] reported that, due to inconsistent results from literature, no clear “gender-violence perpetration” pattern could be established. Hamberger [21] conducted a “gender analysis” about intimate partner violence, finding no difference between males and females in terms of “frequency”, whereas males engage in more severe violent behaviours. Lastly, Desmarais and colleagues [9], pooling data from five studies on individuals with mental disorders from the United States, found that women reported significantly higher rates of violence perpetration than men, suggesting that this finding might reflect, for women in the community, increased opportunity of being violent or the more likelihood to disclose violence-related experiences.

Taken together, these findings suggest that people with personality disorders are at greater risk for perpetuating cycles of violence perpetration, whereas people with mood disorders are more predisposed to be a victim across the life span. In another perspective, we could argue that being a victim of violence in early life plays a role in developing a mood disorder and predisposes to revictimization. Regards to SSOPD, results are more ambiguous, so no comment can be made regarding any specific pathway or pattern of violence.

Although somehow in line with previous literature [8, 10, 11, 42, 45] future research comparing diagnoses, gender and age impact, including larger size samples and using more sophisticated methodological approaches are warranted.

### Limitations

This study presents a number of limitations. The cross-sectional nature of the study design is a major limitation making impossible to confirm any causal association between the variables of interest. As a matter of fact, we could not sustain that suffering from a SMI represents a condition favoring experiences of violence.

A second limitation is the relatively small sample size. Furthermore, most of participants belonged to the same ethnicity and socioeconomic class, so the chance of detecting any effects due to interethnic or social differences played a meaningful role was limited.

Thirdly, the study solely based on KIVS, a clinical interview, whose ratings was not compared with other measurements, such as self-report questionnaires. Higher rates in self-reported victimization and perpetration of violence have been shown in comparison to clinical interview [31], so, in this study, the participants’ ratings could depend on their openness to disclose their experiences to others, not on the truth of the facts.

Moreover, we did not perform an interrater reliability analysis of this clinician-administered interview, so we cannot exclude inhomogeneity in the ratings given by various clinicians. Finally, previous researches based on KIVS failed to consider issues of ethnic diversity since the instrument was administered to Sweden and Italian populations only; therefore, the findings reported cannot be generalized. Even if gender distribution across diagnoses reflects that reported in the literature, this finding could affect results.

Furthermore, we conducted the research in an inpatients psychiatric unit, so that the findings reported cannot be generalized to other clinical or outpatient samples likely affected by less severe disorders. Circumstances related to hospital admission may have rekindled memories of past experiences of violence, above all in early life, and may have influenced the patients’ report.

## Conclusions

This study offers a better understanding of how gender and age could affect violent behaviours. Moreover, our findings may increase the comprehension of the reason why some mental disorders, compared to others, are more associated with the risk of victimization (i.e., mood disorders) or perpetration of violence (i.e., personality disorders). These patterns could have pathophysiological or pathoplastic meaning addressing clinical and diagnostic trajectories and they could interact with other intervening risk factors.

## Data Availability

The datasets generated and analyzed during the current study are not publicly available as they contain individual-level data, but may be available from the corresponding author on reasonable request.

## Abbreviations

ANOVA: Analysis of variance
CATIE: Clinical Antipsychotic Trials of Intervention Effectiveness
IV: Interpersonal violence
KIVS: Karolinska Interpersonal Violence Scale
SMI: Severe mental illness
SSOPD: Schizophrenia spectrum and other psychotic disorders
